# Interaction of a Ti–Cu Alloy with Carbon: Synthesis of Composites and Model Experiments

**DOI:** 10.3390/ma12091482

**Published:** 2019-05-07

**Authors:** Dina V. Dudina, Tomila M. Vidyuk, Michail A. Korchagin, Alexander I. Gavrilov, Natalia V. Bulina, Maksim A. Esikov, Masanari Datekyu, Hidemi Kato

**Affiliations:** 1 Lavrentyev Institute of Hydrodynamics SB RAS, Lavrentyev Ave. 15, 630090 Novosibirsk, Russia; esmax@yandex.ru; 2 Institute of Solid State Chemistry and Mechanochemistry SB RAS, Kutateladze str. 18, 630128 Novosibirsk, Russia; vidyuk@itam.nsc.ru (T.M.V.); korchag@solid.nsc.ru (M.A.K.); gavr_sand@mail.ru (A.I.G.); bulina@solid.nsc.ru (N.V.B.); 3 Novosibirsk State Technical University, K. Marx Ave. 20, 630073 Novosibirsk, Russia; 4 Khristianovich Institute of Theoretical and Applied Mechanics SB RAS, Institutskaya str. 4/1, 630090 Novosibirsk, Russia; 5 Institute for Materials Research, Tohoku University, Aoba Ku, 2-1-1 Katahira, Sendai, Miyagi 980-8577, Japan; datenine.com@gmail.com (M.D.); hikato@imr.tohoku.ac.jp (H.K.)

**Keywords:** Ti–Cu alloys, carbon, in-situ synthesis, ball milling, spark plasma sintering, copper matrix composites, titanium carbide, diffusion

## Abstract

Titanium carbide (TiC), is the most thermodynamically stable compound in the Ti–C–Cu system, which makes it a suitable reinforcement phase for copper matrix composites. In this work, the interaction of a Ti–Cu alloy with different forms of carbon was investigated to trace the structural evolution leading to the formation of in-situ TiC–Cu composite structures. The reaction mixtures were prepared from Ti_25_Cu_75_ alloy ribbons and carbon black or nanodiamonds to test the possibilities of obtaining fine particles of TiC using ball milling and Spark Plasma Sintering (SPS). It was found that the behavior of the reaction mixtures during ball milling depends on the nature of the carbon source. Model experiments were conducted to observe the outcomes of the diffusion processes at the alloy/carbon interface. It was found that titanium atoms diffuse to the alloy/graphite interface and react with carbon forming a titanium carbide layer, but carbon does not diffuse into the alloy. The diffusion experiments as well as the synthesis by ball milling and SPS indicated that the distribution of TiC particles in the composite structures obtained via reactive solid-state processing of Ti_25_Cu_75_+C follows the distribution of carbon particles in the reaction mixtures. This justifies the use of carbon sources that have fine particles to prepare the reaction mixtures as well as efficient dispersion of the carbon component in the alloy–carbon mixture when the goal is to synthesize fine particles of TiC in the copper matrix.

## 1. Introduction

In the area of metal matrix composites, extensive research has been aimed at finding ways of controlling the distribution of reinforcing particles in metal matrices. In comparison with ex-situ processing techniques, synthesis approaches based on the in-situ formation of particles in metal matrices offer more possibilities to control the particle size and particle distribution pattern in the composites [1,2]. 

For copper matrix composites, one of the promising reinforcements is titanium carbide, TiC, which is the most thermodynamically stable compound in the Ti–C–Cu system. TiC–Cu composites have recently been obtained through different processing routes [3,4,5,6,7,8,9,10,11,12]. The synthesis of carbide-containing composite materials from different sources of carbon has attracted attention due to the possibility of controlling the size of the carbide particles and their distribution in the matrices. Sadeghi et al. [11] studied the effect of the carbon source used for the synthesis of in-situ TiC–Cu on the phase formation of the composites and their tribological properties. In that study, carbon nanotubes, graphite, and graphene were used. It was concluded that carbon nanotubes and graphene as carbon sources allow synthesizing finer TiC particles, which considerably improves the tribological properties of the composites. A comparison of the behavior of Ti/multi-walled carbon nanotubes and Ti/carbon black mixtures during ball milling and subsequent thermal explosion was reported by Korchagin et al. [13]. Similar trends were observed in the mixtures in terms of dependence of the characteristic temperatures of thermal explosion on the milling time. However, Ti/nanotube mixtures required shorter milling times to enable the initiation of thermal explosion compared with Ti/carbon black mixtures. This difference was attributed to a higher specific surface area of the nanotubes. Jiang et al. [14] used the aluminum melt reaction method to produce Al–Ti–C master alloys (grain-refining additives to aluminum) from graphite particles and carbon nanotubes as sources of carbon. It was shown that the recovery rate of carbon is higher when nanotubes are used. Jin et al. [15] found that the reaction between carbon nanotubes and titanium in the self-sustaining mode was possible in the presence of unusually high concentrations of metallic diluents due to high reactivity of the carbon nanotubes. A possibility of using nanodiamonds for the synthesis of Al–TiC composite powders by reactive ball milling of Al–Ti–C powder mixtures was demonstrated by Popov et al. [16]. They suggested that the hard particles of nanodiamonds can intensify the mechanical alloying process.

Metal matrix composites obtained by non-equilibrium consolidation of ball-milled mixtures can inherit structural features from the powders and demonstrate unique microstructures unachievable by conventional sintering. One of the modern non-equilibrium methods of obtaining metal matrix composites from powders is Spark Plasma Sintering (SPS) [17]. Fast heating by pulsed current and the application of pressure help achieve densification of metal matrix composite powders within a few minutes [8,18,19]. 

In Ti–Cu alloys, only titanium can form a compound with carbon. Therefore, when carbon combines with titanium upon the interaction of the alloy with carbon, metallic copper is released (provided the amount of carbon is sufficient to fully bind titanium contained in the alloy). Therefore, it is important to identify factors influencing the size of the in-situ formed titanium carbide particles and their distribution in the copper matrix, which is also formed in-situ, when this reactive transformation occurs. It is also necessary to understand whether the location of the carbide particles in the composite obtained via a solid-state route is determined by the location of the carbon particles in the reaction mixture. According to [20], carbon can dissolve in the TiCu intermetallic, which allows one to assume that carbon may diffuse through the alloy layers and cause precipitation of carbides in locations other than the initial locations of the carbon particles in the reaction mixture. 

The goal of the present work was to study the solid-state interactions in the Ti_25_Cu_75_+C system and the formation of in-situ TiC–Cu composites from different sources of carbon. The alloy and the carbon source material were brought in contact by co-milling in a high-energy ball mill; further reaction advancement occurred during the subsequent SPS.

## 2. Materials and Methods 

Ti_25_Cu_75_ ribbons were produced by rapid quenching using the single-roller melt-spinning technique. Master alloys were obtained from titanium and copper (99.99% purity), which were arc-melted in an argon atmosphere. The ribbons were cut into pieces 2–3 mm in length and milled together with carbon black (95% purity, particle size of 100–200 nm, Omsk, Russia) or nanodiamonds (TU84-112-87, diamond content greater than 91%, primary particle size 5 nm, “Altai”, Biysk, Russia) in a high-energy planetary ball mill AGO-2 (ISSCM SB RAS, Novosibirsk, Russia) with water-cooled vials. The carbon black powder and the nanodiamond powder were annealed in vacuum at 800 °C for 60 min before the milling experiments to remove possible volatile components. The vials loaded with powders were filled with argon prior to milling. The milling time of the mixtures was 5 min. The ball/powder weight ratio was 18:1. The amount of carbon black/nanodiamonds added to the alloy corresponded to a molar ratio of Ti/C of 1. A full transformation of titanium contained in the alloy into TiC leads to the formation of the 36% vol TiC–Cu composite. 

The ball-milled mixtures were sintered using a SPS Labox 1575 apparatus (SINTER LAND Inc., Nagaoka, Japan) at a temperature of 900 °C. Graphite dies and tungsten punches with a diameter of 10 mm were used. The die wall was lined with graphite foil; circles of the foil were placed between the sample and the flat ends of the punches. The samples were held at the maximum temperature for 3 min. A uniaxial pressure of 40 MPa was applied. The heating rate in all SPS experiments was 70 °C min^−1^. The temperature during the SPS was measured by a pyrometer (CHINO, Tokyo, Japan) focused on a near-through hole in the die wall.

The phase composition of the samples was investigated by X-ray diffraction (XRD) using a D8 ADVANCE diffractometer (Bruker AXS, Karlsruhe, Germany) with Cu Kα radiation. The lattice parameters of the phases were calculated using PowderCell 2.4 software [21]. The morphology of the powders and the microstructure of the sintered compacts were studied by Scanning Electron Microscopy (SEM) using an UlLTRA 55 microscope (Carl Zeiss, Oberkochen, Germany), a TM-1000 Tabletop microscope (Hitachi, Tokyo, Japan), and a S-3400N microscope (Hitachi, Tokyo, Japan). The X-ray elemental point analysis of selected regions of the polished cross sections was carried out using an Energy Dispersive Spectroscopy (EDS) unit (NORAN Spectral System 7, Thermo Fisher Scientific Inc., Waltham, MA, USA) attached to the S-3400N microscope. The fine structure of the samples was observed by transmission electron microscopy (TEM) using an EM-002B (TOPCON, Tokyo, Japan) microscope working at 200 kV. Ion milling was used to prepare samples of the sintered materials for TEM studies. Vickers hardness of the composites was measured using a Dura Scan 50 hardness tester (EMCO-TEST, Kuchl, Austria) at a load of 0.05 kg. Measurements were made on the polished samples.In order to study the possible diffusion processes though the Ti_25_Cu_75_ alloy/C interface, experiments on sintering of the alloy in contact with graphite were conducted at 700 and 800 °C. Experiments were conducted in a SPS die using graphite foil under a pressure of 40 MPa; the holding time at the maximum temperature was 10 min. Cross sections of the specimens were used for the microstructural studies.

## 3. Results and Discussion

**Figure 1:**
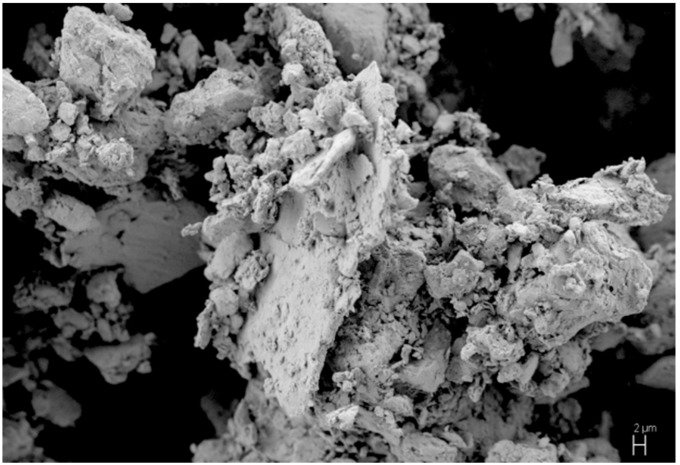
Morphology of the powder agglomerates obtained by ball milling of Ti_25_Cu_75_ alloy ribbons with carbon black.

When carbon interacts with the Ti_25_Cu_75_ alloy, the latter is a source of both the matrix metal and a reactant to synthesize the reinforcing phase. The formation of TiC can potentially occur at any contact of a carbon particle with a particle of the alloy. Figure 1 shows the morphology of the ball-milled composite powder obtained using carbon black. There are flaky elements in the agglomerates, which are the remaining (un-milled) pieces of the ribbons. 

**Figure 2:**
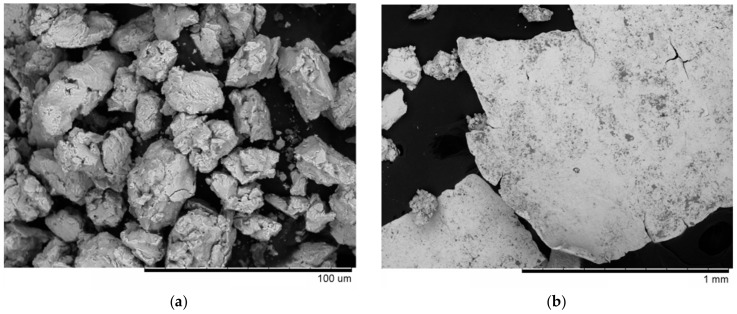
Morphology of the powder agglomerates obtained by ball milling of Ti_25_Cu_75_ alloy ribbons with nanodiamonds (**a**); pieces of ribbons remaining in the product of ball milling (**b**).

The powder obtained by ball milling of the Ti_25_Cu_75_ alloy with carbon black did not contain any millimeter-sized (coarse) fraction. However, in the mixture prepared from nanodiamonds instead of carbon black, the product of milling consisted of a powder and pieces of ribbons not ground into a powder, as shown in Figure 2a,b, respectively. These pieces were separated by sieving and were not used for further sintering. 

**Figure 3:**
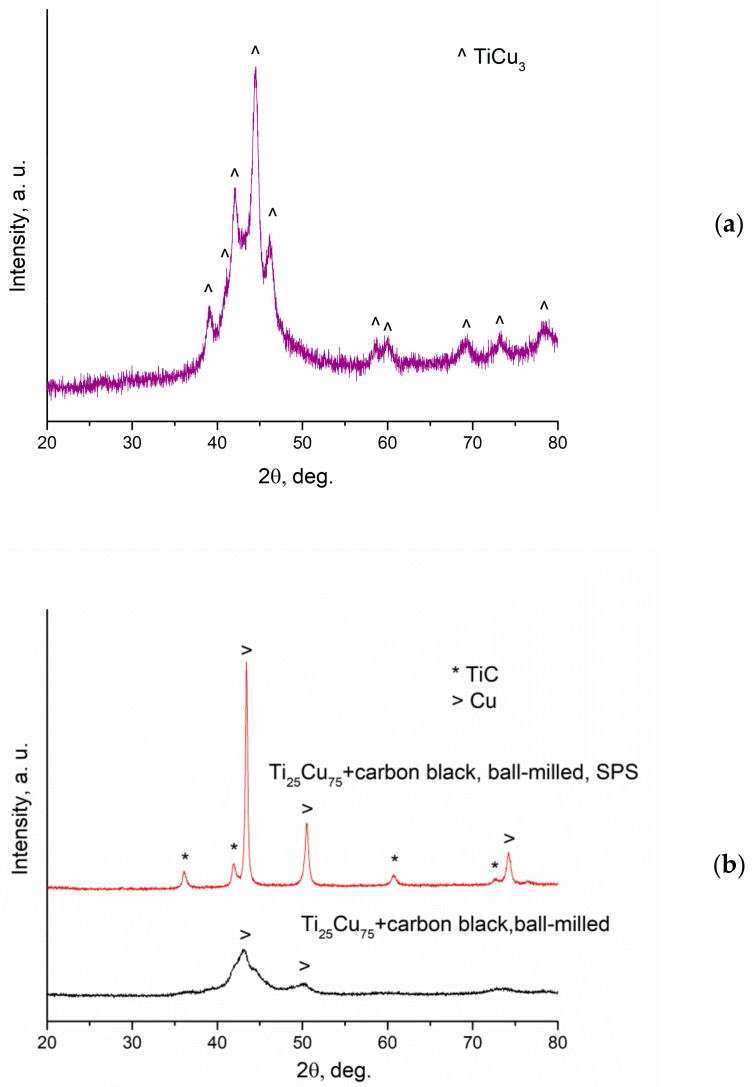

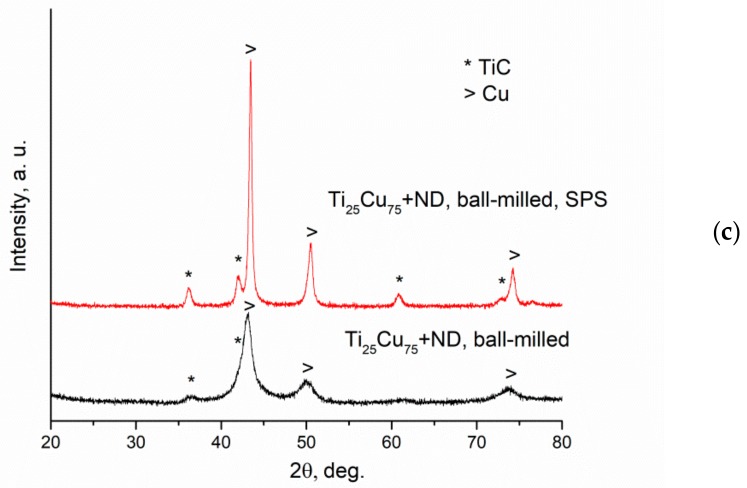
X-ray diffraction (XRD) patterns of the Ti_25_Cu_75_ alloy ribbon (TiCu_3_ PDF card 00-025-316) (**a**); reaction mixture obtained by ball milling of Ti_25_Cu_75_ with carbon black and sintered composite (**b**); reaction mixture obtained by ball milling of Ti_25_Cu_75_ with nanodiamonds (coarse fraction separated) and sintered composite (**c**).

The XRD pattern of the as-spun ribbon is presented in Figure 3a. It is seen that the alloy contains the metastable TiCu_3_ phase and an amorphous phase. The XRD analysis of the ribbon pieces separated from the ball-milled mixture prepared using nanodiamonds as a carbon source showed that the un-milled pieces of the alloy retained the phase composition of the as-spun ribbon.

The XRD patterns of the ball-milled mixtures and corresponding sintered composites are shown in Figure 3b–c. The pattern of the ball-milled mixture prepared using nanodiamonds was recorded after the un-milled ribbon pieces were separated. The XRD patterns of the ball-milled mixtures prepared using carbon black and nanodiamonds differ significantly. The two carbon sources differ by the particle size, specific surface area, and hardness. The surface area of the nanodiamond powder is 360 m^2^ g^-1^ [22], while that of the carbon black powder is only 23 m^2^ g^-1^ [23]. Nanodiamonds reacted with the alloy producing a TiC–Cu product during milling. The formation of TiC leads to the evolution of heat, which does not favor a particle size reduction by brittle fracture of the alloy particles. Worth noting is the fact that, after the milling operation was complete, the vials taken out of the mill started to heat up (being still unloaded). This was an indication of the reaction that had occurred inside the vials. No heating of the vials upon milling completion was observed in the case of mixtures prepared from carbon black. 

**Figure 4:**
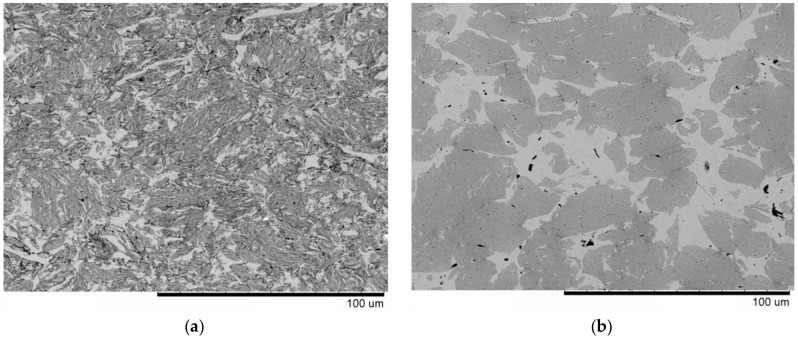
Microstructure of TiC–Cu composites obtained by Spark Plasma Sintering (SPS) of (**a**) the ball-milled mixture of Ti_25_Cu_75_ and carbon black, (**b**) the ball-milled mixture of Ti_25_Cu_75_ and nanodiamonds, from which un-milled pieces of ribbons were separated. The images were taken in the back-scattered electron mode.

**Figure 5:**
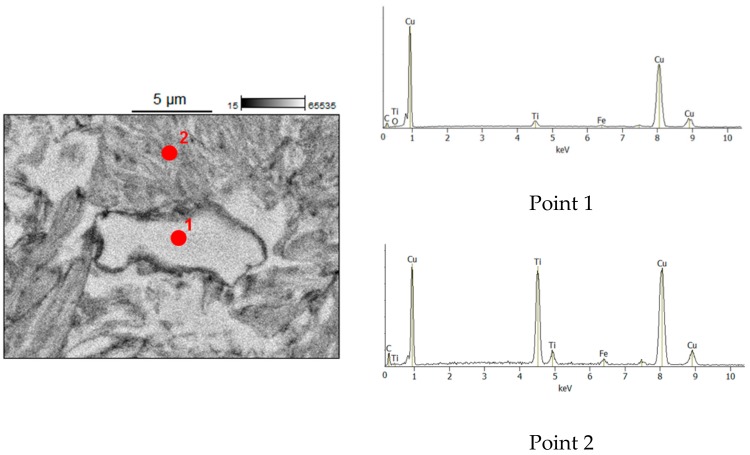
Energy Dispersive Spectroscopy (EDS) analysis of the TiC–Cu composite obtained by SPS of the ball-milled mixture of Ti_25_Cu_75_ and carbon black.

As a coarse fraction of the product of milling (the alloy pieces) was separated from the mixture prepared from the nanodiamonds, the material became enriched with carbon. As can be seen in Figure 4 a,b, the microstructures of the composites obtained using carbon black and nanodiamonds bear similarities in the sense that composite TiC–Cu regions are intermixed with Cu-rich regions. The EDS analysis (Figure 5) confirmed a higher concentration of copper in the bright regions as compared with the dark regions of the composite. However, the Cu-rich regions were larger in the composite produced from nanodiamonds. Furthermore, the composite TiC–Cu regions in the material obtained from nanodiamonds showed fewer bright strips, which implies that those regions contained less copper than the same regions in the composite produced from carbon black. The relative density of the composite obtained using carbon black was 95.2%. For the composite obtained from nanodiamonds, it was not possible to calculate the relative density, as the exact composition (and, hence, the theoretical density) of the composite containing an excess of carbon was not determined.

The lattice parameters of copper and titanium carbide were calculated from the XRD profiles of the sintered composites using Rietveld analysis in Powder Cell 2.4 software, as shown in Table 1. The values of the lattice parameter of titanium carbide in the composites suggest carbon deficiency of the compound. According to [24], these lattice parameter values correspond to the TiC_0.72_ stoichiometry. A deviation of the composition from the stoichiometric TiC implies that some carbon remains in the copper matrix in the form of particulate inclusions, which may serve as reinforcements together with the titanium carbide particles. A slightly larger lattice parameter of copper in the composites in comparison with pure copper indicates the formation of solid solutions of titanium in copper. The carbon deficiency and the presence of titanium dissolved in copper are beneficial for the formation of the matrix/reinforcement interface in these composites. As was noted by Zarrinfar et al. [3], wetting of titanium carbide particles by molten copper is enhanced by a reduction in the C/Ti ratio in the carbide and the presence of Ti in molten Cu.

If one assumes diffusion of titanium towards the alloy/carbon interface, the thickness of a Ti-depleted layer can be estimated as

*L* = (*Dt*)^1/2^, (1)

where *D* is the diffusion coefficient of titanium in the alloy, and *t* is the time (for calculations, the diffusion coefficient of titanium in copper was used [25]). Calculations show that holding the alloy/carbon system at 900 °C for 3 min produces a Ti-depleted layer about 5 μm thick. Consequently, during sintering, particles of the alloy several micrometers in size could be depleted of titanium as a result of diffusion of the latter to the particle boundaries and further chemical reaction with carbon. In our previous work, we found that phase transformations in the melt-spun Ti_25_Cu_75_ alloy ribbons upon rapid heating lead to the formation of metallic copper, among other (intermetallic) phases [18], which agrees with the observation of the fcc phase in the Ti_25_Cu_75_ alloys of non-equilibrium structure [26]. So, in [18], the formation of copper matrix regions free from TiC particles in the sintered composites was attributed to the transformation of the alloy itself. The structural analysis conducted in this work showed that, during sintering, the depletion of pieces of the alloy ribbons remaining in the ball-milled mixture of titanium can occur through the outward diffusion of titanium caused by its interaction with carbon at the interface. Thus, the outward diffusion of titanium presents another mechanism of the formation of Cu-rich regions in the composites.

**Figure 6:**
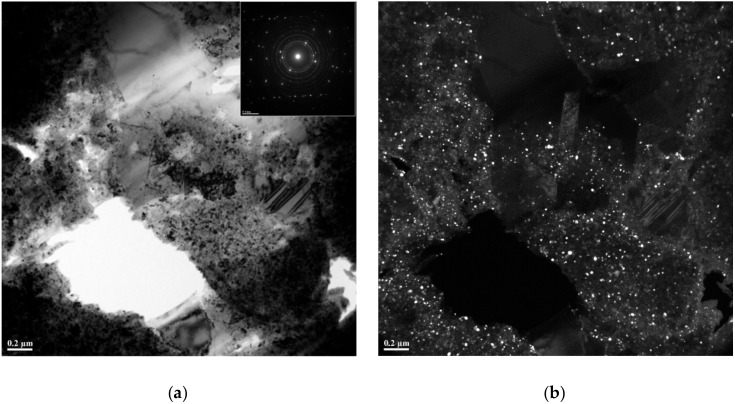
Transmission Electron Microscopy (TEM) bright-field image of the TiC–Cu composite obtained by SPS of the ball-milled mixture of Ti_25_Cu_75_ and carbon black and selected-area electron diffraction pattern (**a**); dark-field image taken in the TiC (111) reflection (**b**).

Results of SEM/EDS carried out on the composites (Figure 4 and Figure 5) fully agree with those of TEM studies. Figure 6a shows a TEM bright-field image of the TiC–Cu composite obtained by SPS of the ball-milled mixture of Ti_25_Cu_75_ and carbon black and a selected-area electron diffraction pattern. A corresponding dark-field image taken in the TiC (111) reflection is shown in Figure 6b, confirming that the composite contained TiC particles 10–20 nm in size distributed in the copper matrix. In the TEM images, Cu-rich areas were also observed in the structure of the composite. 

Vickers hardness of the TiC–Cu composite obtained using carbon black was measured in two directions by making indents on planes that were parallel and normal to the pressing direction during SPS. The obtained values of hardness were 440 ± 20 HV and 450 ± 70 HV, respectively. The high hardness of this material was due to its high relative density and a composite structure containing very fine TiC particles. 

**Figure 7:**
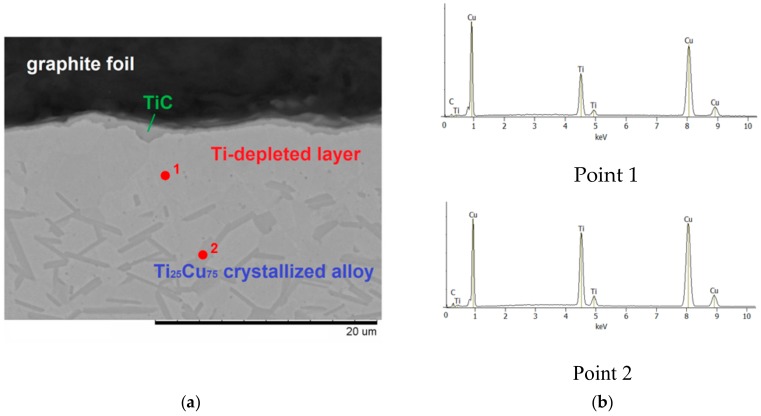

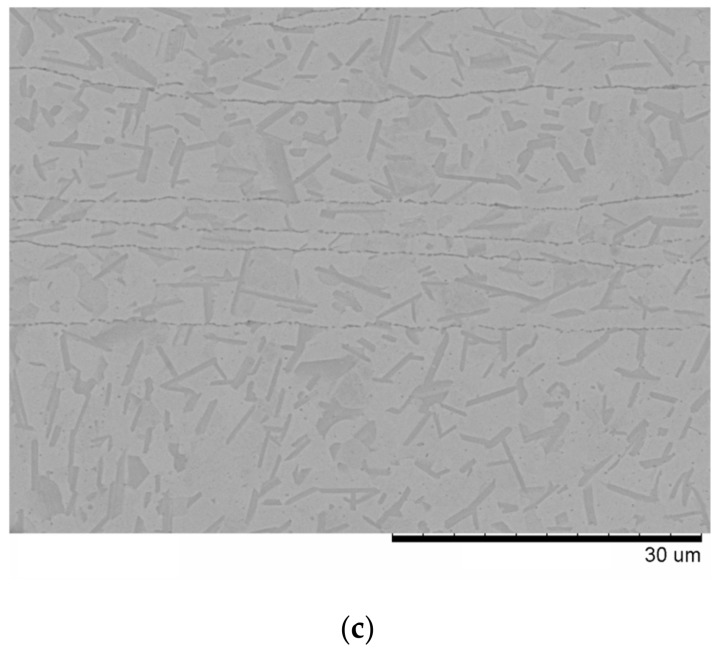
Cross-section of the graphite foil/Ti_25_Cu_75_ ribbon specimen processed by SPS at 800 °C for 10 min: graphite/alloy interface (**a**); results of the EDS analysis (**b**); center of the specimen—microstructure of the crystallized alloy (**c**). The images were taken in the back-scattered electron mode.

The interaction of metals and alloys with carbon during sintering has been the subject of recent studies [27,28,29,30]; however, phase and microstructure changes occurring at the Ti–Cu alloy/carbon interface have not been reported. To understand the details of the evolution of the alloy’s microstructure when the alloy comes in contact with carbon, model experiments were conducted, in which ribbons of the alloy were sintered in contact with graphite foil under pressure. The results of the experiments conducted at 800 °C are reported below. The interface between the alloy and graphite is shown in Figure 7a, while results of the point EDS analysis are given in Figure 7b. It can be seen that the alloy layer adjacent to the interface was free from crystallites rich in titanium (elongated grains showing dark contrast in the image). This indicates that titanium atoms diffused to the alloy/graphite interface and reacted with carbon to form a titanium carbide layer, but carbon did not diffuse into the alloy. A thin layer of TiC was seen at the interface of the graphite foil and the alloy. For comparison, a cross-sectional view of the interior of the specimen consolidated from the ribbons is shown in Figure 7c, which demostrates the structure of the crystallized alloy). 

**Figure 8:**
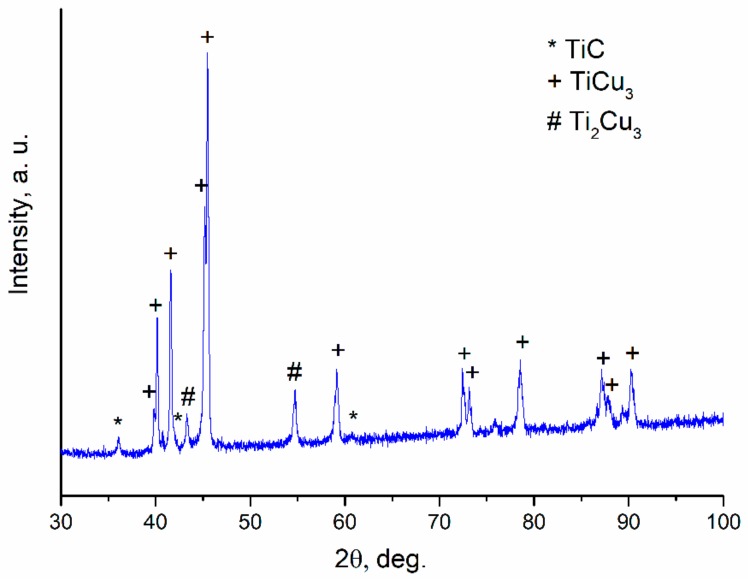
XRD pattern of the specimen obtained by SPS of Ti_25_Cu_75_ ribbons in contact with graphite foil (TiCu_3_ PDF card 00-007-108). The pattern was taken from the flat end of the disk-shaped specimen, from which the graphite foil was carefully removed.

A XRD pattern was taken from the flat end of the sintered graphite foil/Ti_25_Cu_75_ specimen after the graphite foil had been carefully and almost completely removed. A careful operation of foil removal was needed to preserve the layer formed by reaction diffusion. As can be seen in Figure 8, the amorphous part of the alloy crystallized upon sintering. The sintered alloy was TiCu_3_ intermetallic with a structure different from that of the intermetallic phase in the as-spun alloy. Reflections of TiC could also be found on the pattern. At a SPS temperature of 700 °C, a similar effect, Ti-depletion of the layer adjacent to the graphite foil, was observed; however, the layer was thinner. These results are quite different from those obtained in [29,30], in which carbon from the graphite foil was found to diffuse into the alloy during SPS. In that case, the alloy was based on a Ni(W) solid solution obtained by mechanical alloying. Owing to a high solubility of carbon in nickel and a defect crystalline structure of the ball-milled material, carbon was able to diffuse into the sintered material, which caused the formation of WC and Ni_2_W_4_C carbide particles at large distances from the initial alloy/graphite interface. In the case of sintering of the Ti_25_Cu_75_ ribbons in contact with graphite, no evidence of diffusion of carbon through layers of the alloy was detected. This result is in agreement with observations reported by Lloyd et al. [31], who obtained Cu–Ti/carbon fiber composites by hot pressing Cu-coated nanofibers mixed with a Ti powder and found TiC layers covering some carbon fibers. The transport of titanium to carbon was by means of diffusion of the former through the copper layers. Carbon nanofibers located at distances greater than the diffusion distance of titanium in copper under conditions of hot pressing from any titanium particle did not have a TiC layer.

## 4. Conclusions

In this work, solid-state interactions in the Ti_25_Cu_75_+C system were studied using different sources of carbon. A synthesis route of TiC–Cu composites through ball milling of the Ti_25_Cu_75_ alloy with a carbon source (carbon black or nanodiamonds) was evaluated from a perspective of forming fine particles of TiC in the copper matrix. The following conclusions were drawn:

(1) The ball milling outcome of mixtures of Ti_25_Cu_75_ ribbon with C depends on the nature of the carbon source; mixing is more efficient in the case of carbon black than in the case of nanodiamonds;
(2) During sintering, at Ti–Cu alloy/carbon contacts, titanium atoms diffuse to the alloy/graphite interface and react with carbon to form a titanium carbide layer; no evidence of carbon diffusion into the alloy was obtained;
(3) The distribution of TiC particles in the composite structures obtained via reactive solid-state processing of Ti_25_Cu_75_+C follows the distribution of carbon particles in the reaction mixtures; in order to obtain a uniform distribution of nanoparticles of TiC in the copper matrix, carbon source nanoparticles should be thoroughly mixed with the alloy.

## Figures and Tables

**Table 1:** Lattice parameters of the phases of the sintered TiC–Cu composites.

Reaction Mixture Processed by Ball Milling and SPS	Cu Lattice Parameter ^1^, Å	TiC Lattice Parameter ^2^, Å
Ti_25_Cu_75_+carbon black	3.620	4.321
Ti_25_Cu_75_+nanodiamonds	3.623	4.315

^1^ Lattice parameter of pure Cu is 3.615 Å; ^2^ Lattice parameter of stoichiometric TiC is 4.327 Å.
